# Progress in Surgery

**Published:** 1897-08-21

**Authors:** 


### PROGRESS IN SURGERY.

### ORTHOPÆDIC PROGRESS.

**Spinal Caries: Forcible Reduction of the Curve.**—Calot[1] has operated upon thirty-seven cases of curvature of the spine, and has forcibly corrected the curve so soon as it appeared. The method is as follows: The patient is placed under an anæsthetic; while four assistants pull on the upper and lower extremities of the spinal column, another assistant pulls at the head. The surgeon himself, standing over the spine, presses very forcibly indeed on the projection; this is felt to yield sometimes with a distinct crack or snap, which is apt to be rather alarming; in other cases it yields gradually. The patient is then suspended from an apparatus which takes firm grip of the head and beneath the axillæ. In that position—that is the erect position—plaster of Paris is applied. Should the curve not come out entirely at the first setting, the plaster of Paris is taken off in a couple of weeks' time, and forcible reduction is again tried. Sometimes, however, it happens, according to Calot, that there is a posterior wedge of bone which prevents the vertebral column from being straightened; this he has excised in two cases. It would appear at first sight as if such forcible extension of the spinal column must be extremely dangerous, but it seems as if experience disproves this. Calot does not appear to have had any unsatisfactory results, such as paralysis or abscess, nor has the personal experience of the writer been unfavourable in these respects.

**Scoliosis; The Value of an Adapted Bicycle in the Treatment of this Affection.**—Dr. Otto G. T. Kiliani[2] contributes an interesting and suggestive article on this subject. The essence of his article is that, if a handle-bar is lowered on one side and if the saddle be made of two lateral halves, it is possible by suitably arranging these parts of the bicycle to aid muscular action in correcting a curve. The article is fully illustrated by photographs which show that his suggestion is of practical value, but the writer hesitates to speak of the results until he has had more experience in the matter, as at present he has only six patients under treatment. Briefly, the advantages of his line of treatment are as follows: Firstly, a good many of the patients to be treated are in possession of a bicycle; even for those who do not own one the expense is relatively slight in comparison with that of other orthopædic apparatus. Secondly, it affords a physical exercise to which the patients will take kindly, and which they will therefore carry out faithfully. This is a decided point of advantage, as the execution of gymnastic exercises always demands unusual patience and perseverance on the part of both patient and physician. Finally, he describes a simple device by which either handle-bar can be adjusted in any desired position. The handle-bar is sawed apart in the middle, and the two parts are adjusted by means of a screw and thread, fitting one into the other (the two necessary pieces having been soldered into the tubes). The circumference of the two pieces is grooved, and held in place by a wedge with corresponding teeth, the latter being tightened by a screw with nut fastening the jaws of the head, pointing towards the rider. The thread mentioned will allow the lowering of either side of the handle-bar to any degree desired, while the two halves of the handle-bar will still be held firmly together. The wedge with its corresponding teeth will hold all the parts absolutely firm when pressed in against the handle-bar by tightening the screw and nut.

**Congenital Defects of the Long Bones.**—Dr. B. E. McKenzie[3] contributes what is perhaps the most exhaustive article on this subject which has hitherto appeared in surgical literature. He discusses the various long bones seriatim. Speaking of the tibia, he says that in the cases hitherto reported the condyles of the femur have been normally developed, and there was a joint surface on the outer condyle for articulation with the fibula which was generally larger than normal. Forty-five cases of congenital defect of the fibula are reported by Hoffa, and recently two cases were shown at the meeting of the British Orthopædic Society in London. When the fibula is absent the knee-joint is generally normal, but the external lateral ligament may be absent, or the crucial ligaments, or the patella. The foot assumes a position of valgus, and occasionally some of the toes are wanted. Partial defect of the radius occurs but seldom. Dr. McKenzie then details some cases of his own, and speaking of those peculiar cases in which there is an angular curvature of the tibia existing at birth, with a distinct umbilication or dimple of the skin at the most prominent part of the curve, he suggests that such an appearance indicates pre-natal compound fracture. In cases of club-hand, where the radius is absent, the functions of the fingers are more perfect towards the ulna border of the hand.

**Congenital Displacement of Hip.**—A. H. Tubby[4] contributes a long article to the *Lancet* on "The Present Aspect of the Treatment of Congenital Displacement of the Hip." After briefly reviewing the condition of the parts, and more particularly insisting upon the varying shape of the head of the femur, the absence, shortening, or misdirection of its neck, the alterations in the capsule of the joint, and the contraction in the adductor muscle, he then discusses the various lines of treatment. These are three: Extension; operative measures; forcible reduction. He states that in some cases extension, prolonged for 18 months to two years, has proved efficacious to children under four years of age, but not older. Lorenz's and Hoffa's operations are then discussed, and it is pointed out that both are unsatisfactory, but Lorenz's less so than Hoffa's. The most promising method is that of Paci as modified by Professor Lorenz. This consists in reducing the hip by manipulation as in traumatic dislocation, dividing the contracted adductors if need be, and then placing the limb up in the fully abducted position in plaster, and allowing the patient to walk by means of crutches, having placed a patten on the foot. So far this method has proved eminently satisfactory.

On the same subject, G. R. Elliott[5] describes a case, and makes remarks on the new non-cutting operation of Lorenz. His paper is fully illustrated by skiagrams and by diagrams showing the essential points in the manœuvres as advocated by Paci and Lorenz.

**Congenital Absence of Patellæ.**—A. T. Bristow,[6] while examining a child affected with congenital talipes equino-varus, noticed that a luxation of the knees could be produced with great ease. Closer inspection showed that the knee-joints immediately became dislocated on bringing the axes of the femur and tibia into line. Both patellæ were found to be absent. The tendons of the two quadriceps, instead of being thick and strong, were very thin, and were spread out over the front of the joint, seeming to be attached in part to the condyles of the femora, as well as to the tibiæ and fibulæ. It is noticed by those who have considerable experience of these cases that often in the course of time, and generally about the age of four years, the patella begins to develop, but frequently there is considerable hyperextension of the knees left. In every case, so far as the writer's experience shows, some other congenital malformation has been present.

**Tendon - Grafting and Muscle - Transplantation for Deformities following Infantile Paralysis.**—Dr. S. E. Milliken[7] gives supplementary notes of several cases which he had described previously.[8] He says that his past year's experience has brought forth results beyond the expectation of the most ardent advocate of this comparatively new treatment. Since February 14th, 1894, he has performed fourteen operations upon nine patients afflicted with various degrees of deformity due to infantile paralysis. Of this number, five of the patients required one operation, three patients two operations each, and in only one case were three tendon - graftings permitted. To give examples, partial or complete transplantation of the sartorius muscle into the sheath of the paralysed quadriceps extensor of the thigh; grafting of the extensor proprious pollicis to the paralysed tibius anticus; attachment of the gastrocnemius to the paralysed peroneus longus and brevis; attachment of the tibialis anticus to the paralysed extensor longus digitorum. His conclusions as to the value of the operations are as follows: 1. Infantile paralysis in the majority of instances attacks groups of muscles or an individual muscle of a group. 2. Operative interference should be practised with the hope of re-establishing the symmetry of the limb, and can be accomplished in one of two ways—(*a*) when the whole group is paralysed, a healthy muscle with the proper origin must be transplanted, and given the insertion of the paralysed group; (*b*) when only part of the group is involved, tendon-grafting should be performed—that is, making one or more muscles do the work of those paralysed. 3. Animal suture material, preferably kangaroo tendon, should be employed on the tendons and muscles and in the closure of the sheath. As this material requires 21 days for absorption, it will usually be found that at the expiration of that time perfect union will have been obtained. 4. The skin wounds should be closed with interrupted catgut, and the sealed dressing of cotton collodion applied. 5. Perfect immobilisation of the limb can best be obtained by the plaster of Paris splint. 6. The best results of such operative procedures can be obtained only in young subjects, so as to take advantage of the natural growth of an otherwise undeveloped muscle, upon which we place additional work, as it would be unreasonable to expect a man who has led a sedentary life up to the age of 50 to assume at that time the arduous labour of a mechanic.

**Osteo-Plastic Resection of the Tarsus.**—Negretto[9] describes a new operation applicable in cases where the disease is confined to the posterior part of the foot, and where it is desired to save as much of the foot as possible. The operations of Vladimirow and Mikulicz for this purpose result in the foot being fixed in a position of talipes equinus. The author's operation allows the patient to walk on the sole of the foot, which is fixed at right angles to the leg. This result is brought about by resecting portions of bone from the tibia and fibula, and from the dorsal face of the navicular, cuboid, and second and third cuneiform bones, the two surfaces (after previous removal of the diseased os calcis and astragulus) being then united together. The foot is necessarily shortened by this method, but the author claims that less mutilation occurs, tenotomy of the flexor tendons of the sole of the foot is unnecessary, the operation is shorter and more conservative, and, above all, the foot is so placed that, instead of walking on the toes, as after Mikulicz's operation, the patient is able to use the sole of the foot as before. In the single case in which the author has put his method into practice the results were decidedly satisfactory.

**Paralytic Club-Foot: Treatment by an Osteo-Plasty in Combination with Arthrotomy.**—Jenardi[10] reported two interesting cases in which he produced excellent results by the implantation of a fragment of denuded bone between the astragulus and tibia, thus producing a bony ankylosis.

He was led to this procedure by finding, on repeating an operation that had been unsuccessful, that there was a wedge-shaped space between the astragulus and tibia which had been filled only by connective tissue, and consequently did not resist the counter-pressure after the joint was released from the bandage. Reasoning that the result was similar to that of ununited fractures, where the bones are not in close apposition, he determined to induce bony ankylosis by filling in the space with freshly denuded bone. This was done, using a fragment of child's bone which had just been resected. The fragment was held in place by an iron wire suture, which ulterated out later, with a small sequestrum. The author made the observation at that time that the new bone was penetrated by many arterial capillaries, showing that it acted as the skeleton over which the new bone grew, and was not of itself permanent.

[1] La Semaine Médicale, December 23rd, 1896. [2] New York Med. Record, October 31st, 1895. [3] New York Med. Journ., February 20th, 1897. [4] Lancet, May 1st, 1897. [5] New York Med. Record, May 29th, 1897. [6] Lancet, January 23rd, 1897. [7] New York Med. Record, November 28th, 1896. [8] Ibid, October 26th, 1895. [9] Therapeutic Gazette October 15th, 1896. [10] Cent. Für. Chir., March, 1896.

